# Early indicators of microbial strain dysbiosis in the human gastrointestinal microbial community of certain healthy humans and hospitalized COVID-19 patients

**DOI:** 10.1038/s41598-022-10472-w

**Published:** 2022-04-21

**Authors:** Hyunmin Koo, Casey D. Morrow

**Affiliations:** 1 Department of Genetics, Hugh Kaul Precision Medicine Institute, University of Alabama at Birmingham, Birmingham, AL USA; 2 Department of Cell, Developmental and Integrative Biology, Hugh Kaul Precision Medicine Institute, University of Alabama at Birmingham, Birmingham, AL USA

**Keywords:** Microbiome, Data mining

## Abstract

Dysbiosis in the human gastrointestinal microbial community could functionally impact microbial metabolism and colonization resistance to pathogens. To further elucidate the indicators of microbial strain dysbiosis, we have developed an analytic method that detects patterns of presence/absence of selected KEGG metabolic pathways for a selected strain (PKS). Using a metagenomic data set consisting of multiple high-density fecal samples from six normal individuals, we found three had unique PKS for important gut commensal microbes, *Bacteroides vulgatus* and *Bacteroides uniformis,* at all sample times examined. Two individuals had multiple shared PKS clusters of *B. vulgatus* or *B. uniformis* over time. Analysis of a data set of high-density fecal samples from eight COVID-19 hospitalized patients taken over a short period revealed that two patients had shared PKS clusters for *B. vulgatus* and one shared cluster for *B. uniformis*. Our analysis demonstrates that while the majority of normal individuals with no *B. vulgatus* or *B. uniformis* strain change over time have unique PKS, in some healthy humans and patients hospitalized with COVID-19, we detected shared PKS clusters at the different times suggesting a slowing down of the intrinsic rates of strain variation that could eventually lead to a dysbiosis in the microbial strain community.

## Introduction

Investigating the stability of the gut microbial community is important due to the growing realization of the role of these communities in human health^1,2^. Previous studies have shown that the taxonomic composition that the human gut microbial community is relatively stable over time^3,4^. More recent studies have used a more in-depth analysis consisting of metagenomic DNA sequencing of microbial communities in conjunction with new informatics to establish the gut microbial community consists of a consortium of microbial strains^5–7^. In a previous study, we used metagenomic DNA sequencing analysis with a Window-based Single Nucleotide Variant (SNV) Similarity (WSS) program to assess the strain relatedness of the microbes in two separate samples from the same individual^5^. Using paired samples from the data set from the Human Microbiome Project (HMP)^8^, we established cut-off values for the WSS scores that can discern between related and unrelated samples to demonstrate that microbial strains are unique to the individual^5,9,10^. In general, the dominant fecal microbial strain communities are stable over time although the extent of temporal relatedness of microbial strains is individual specific^11^.

In follow-up studies using the WSS, we have shown that the gut microbial strain community can be influenced by overt changes in the gut environment^11,12^. For example, we have seen that a drastic change in the environment that occurs as a result of antibiotics, oral drugs, or physical disruption of the gastrointestinal tract can result in the appearance of new gut microbial strains in certain individuals^12–15^. However, we have not seen instances of the change of the dominant strain in normal individuals over time without defined disruptions. A reason for this could be due to the resiliency of the gut microbial strain community that corrects for the short-term changes. Although we have not identified instances where strain change occurred without disruption of the gut environment, we know that this occurs from previous studies on the gut microbe strain stability from sets of twins^11^. In this study, we found strain sharing was dependent on the time of cohabitation. The strain differences from twins that had been separated for longer times were attributed to environmental differences. A limitation of these studies though was the time in between sampling that would have missed any subtle changes in the gut microbial community that would have signaled a forthcoming strain change.

In the current study, we have sought to further define the dynamics of gut microbial strain variation. We have used several public data sets from a high density of longitudinal collection of fecal samples^16,17^. Our study combines strain tracking and a new method to analyze microbial strain variation based on comparing the patterns of presence/absence of selected KEGG metabolic pathways for a selected strain (*herein* PKS). We demonstrate that most healthy individuals from the HMP data set and a second high-density data set with shorter time intervals have individuals with shared and unique PKS overtime for important gut commensal microbes, *Bacteroides vulgatus and Bacteroides uniformis*. Furthermore, analysis of a recently described data set of longitudinal fecal samples from hospitalized patients with COVID-19 revealed two of eight patients had clusters of shared PKS that did not disappear over time^17^. The PKS clusters in some individuals suggest a dysbiosis in which a slowing down of the intrinsic rates of change occurs in the microbial community that could be an early warning signal for strain replacements leading to functional alterations of metabolism and colonization resistance^18–20^.

## Results

### Processes of PKS analysis

In this study, we developed the PKS analysis to further our investigation into the dynamics of strain variation in the human gut microbial community. To do this, we used the original sequence reads from the HMP data set^8^ in which we used the WSS analysis to show related microbial strains in samples taken at two different times^5^. Using sample pairs from 41 different individuals, we determined the PKS for *B. vulgatus* and *B. uniformis* strains in each sample pair. We have focused our analysis on *B. vulgatus* and *B. uniformis* since these commensal microbes are present in high relative abundance in the human microbial community and possibly represent a keystone species^9,21–23^. From the 39 pairs of 41 that had related *B. vulgatus* strain in the HMP, a total of 25 KEGG pathways showed that 18 related pairs (46.1%) had no differences in PKS (Supplementary Fig. 1 and Supplementary Table 1). For *B. uniformis*, 35 pairs of 41 that had related *B. uniformis* strain in the HMP, a total of 40 KEGG pathways showed that 13 related pairs (37.1%) had no differences in PKS.

One of the features of the PKS analysis was to determine the presence/absence of selected KEGG metabolic pathways between the related dominant microbial strains from paired samples. To further employ the PKS analysis then, we determined if the numbers of pairs with differences in the PKS would change by reducing the number of sequences read that were analyzed. To do this, we rarefied our analysis by randomly subsampling each sample from the HMP data set at 2.5, 5, and 10 million sequence reads and then conducting a WSS analysis followed by a PKS analysis. For the 2.5 and 5 million reads, we repeated the random subsampling process two more times to detect any variations in the WSS score or PKS (Supplementary Fig. 1 and Supplementary Table 1). From this analysis, we found a leveling off of the zero pathway difference percentage between 2.5 and 10 million reads, suggesting the analysis of the related *B. vulgatus* strains was reached at this read number. Based on this analysis, we selected 5 million reads for the subsampling process to establish a list of KEGG pathways (including 23 pathways) for *B. vulgatus* and to apply for the same reads number when other data set are used for the PKS analysis. Similar to *B. vulgatus* strain, we have selected 5 million reads for *B. uniformis* strain to run the PKS analysis (Supplementary Figs. 1 and 3).

**Figure 1:**
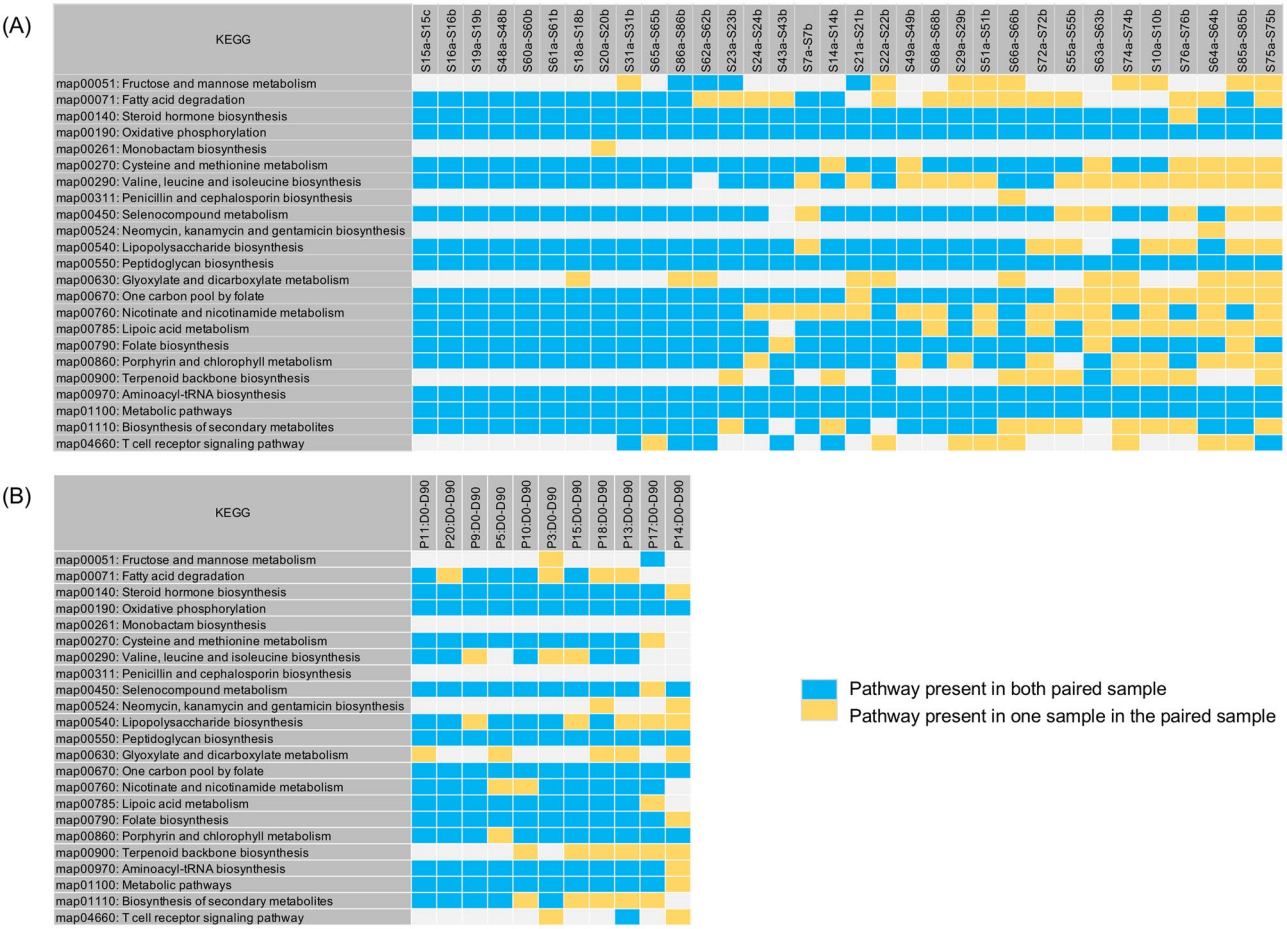
PKS results for healthy individuals. A total of 23 KEGG pathways were used to investigate a pattern of presence/absence of metabolic pathways for *B. vulgatus.* The summarized PKS result of the 23 KEGG pathways per individual related pair from (**A**) HMP^8^, and (**B**) Raymond et al.^24^ data sets were grouped into different color boxes (colors scheme presented in the figure). Each column in the table indicates an individual’s paired samples and matches the number shown in Supplementary Table 2.

From the 5 million sequences subsampled from the HMP data set, using the WSS analysis we found that an average value of 34 individual pairs had related *B. vulgatus* strains (Supplementary Fig. 1 and Supplementary Table 1). We next examined related pairs for differences in PKS from a total of 23 KEGG pathways that are specific for *B. vulgatus.* From one of the repeat sets, we found that 6 of the 33 sample pairs (18.1%) had 0 changes in the compared KEGG pathways for *B. vulgatus* (Fig. 1A). The remaining 27 individual pairs (81.8%) showed a range from 1 to 13 changes in the compared KEGG pathways with no shared patterns (Fig. 1A). Overall, an average value of 6 of the 34 sample pairs (17.6%) had 0 changes in the compared KEGG pathways and the remaining 28 pairs (82.4%) had no shared patterns for *B. vulgatus* (Supplementary Fig. 1 and Supplementary Table 1). For *B. uniformis*, we found that an average value of 3 of the 24 sample pairs (12.6%) had 0 changes in the compared KEGG pathways and the remaining 21 pairs (87.4%) had no shared patterns for *B. uniformis*.

Our analysis of the HMP data set has shown differences in PKS between related individual pairs for a shared strain. However, the HMP data set consisted mostly of pairs with time frames within 1 year (with some even longer)^8^. To better characterize the PKS analysis, we used a second data set consisting of healthy individual pairs that had been sampled at day 0 and day 90^24^. We have previously confirmed that individual pairs had a WSS score above the cut-off value indicating that the pairs were related^14^. Using sequences randomly subsampled to 5 million reads, we found that 11 pairs had related *B. vulgatus* strain. A PKS analysis of each pair found that none of the 11 pairs had shared PKS patterns (Fig. 1B).

### PKS analysis of high-density longitudinal samples from healthy individuals

**Figure 2:**
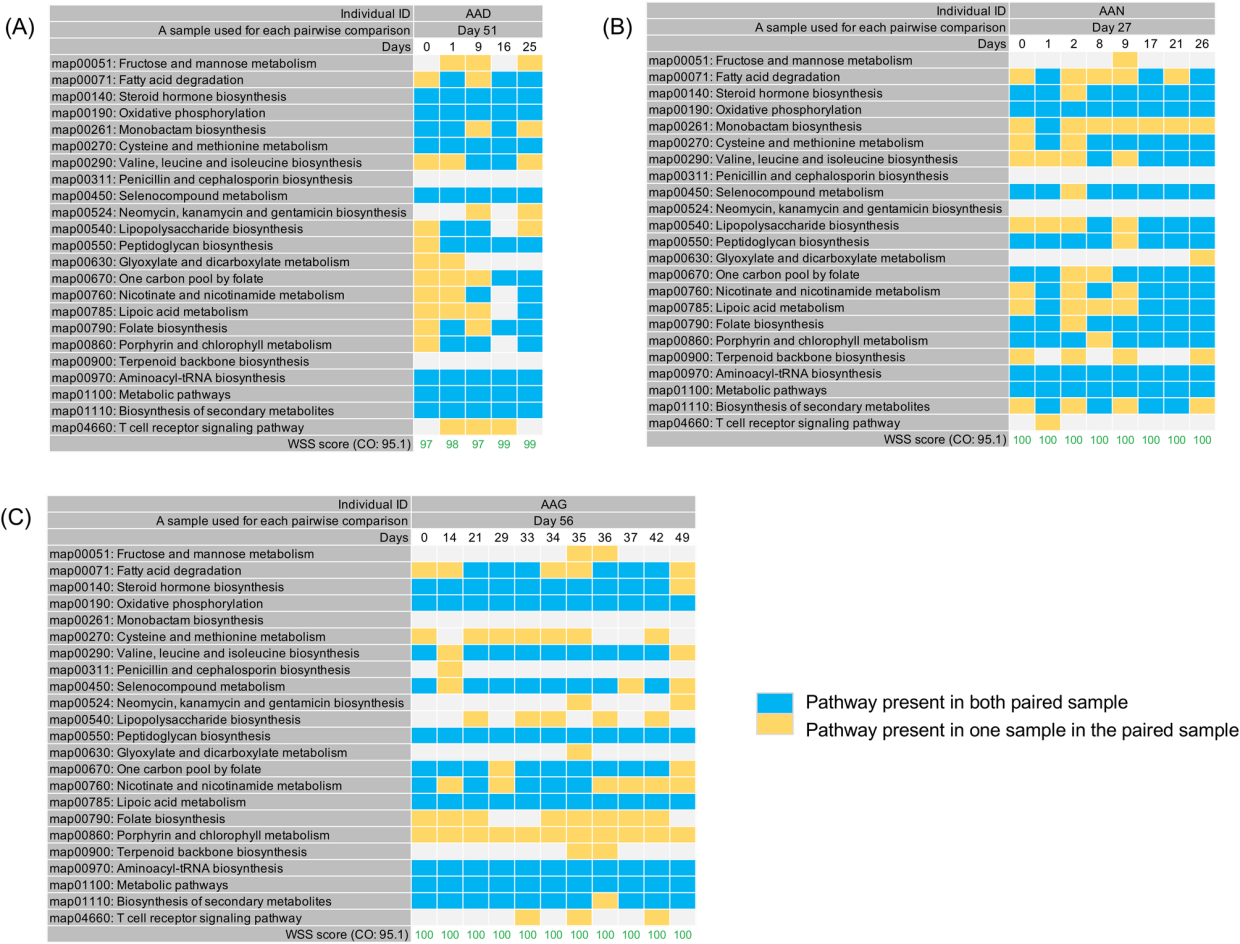
PKS analysis from high density sampling of healthy individuals. A total of 23 KEGG pathways were used to examine the presence/absence of KEGG metabolic pathways for *B. vulgatus* by comparing each individual’s last day sample to every possible pair of the same individual’s samples. The WSS scores and cut-off value for *B. vulgatus* are noted. All samples from the three individuals, including (**A**) AAD, (**B**) AAN, and (**C**) AAG were previously collected by Fukuyama et al.^16^. The summarized PKS result per individual was grouped into different color boxes (The color scheme for presence and absence are the same as in Fig. 1). Each column in the table represents individual ID, a sample used for each pairwise comparison, and days (Detailed sample information shown in Supplementary Table 2).

**Figure 3:**
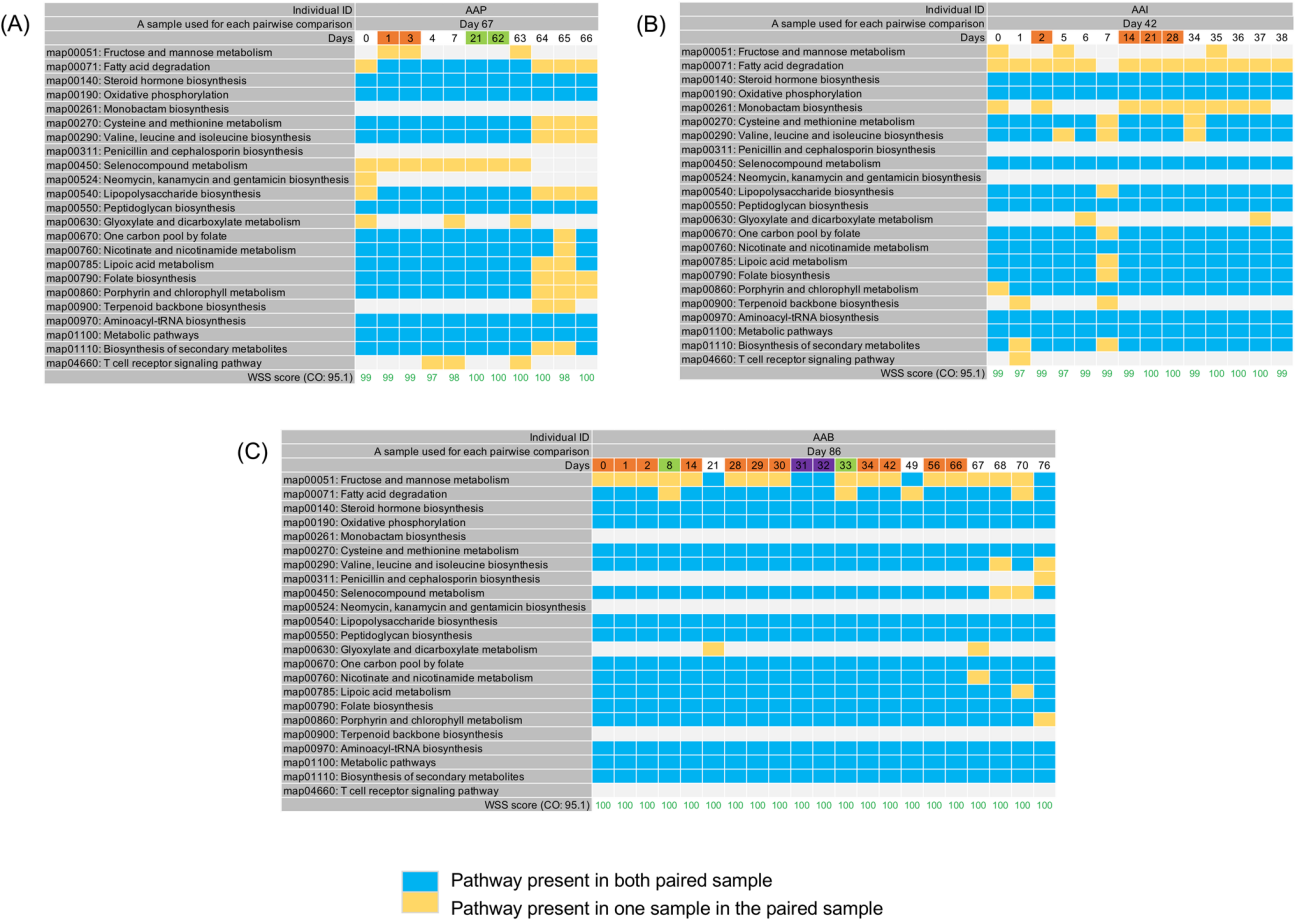
Shared PKS results from high density sampling of three healthy individuals. A total of 23 KEGG pathways were used to observe a pattern of presence/absence of KEGG metabolic pathways for *B. vulgatus* and the presence or absence of each KEGG pathway was examined by comparing each individual’s last day sample to every possible pair of the same individual’s samples. All samples from the three individuals, including (**A**) AAP, (**B**) AAI, and (**C**) AAB were previously collected by Fukuyama et al.^16^. The shared PKS result per individual was depicted by different colored boxes. Each column in the table represents individual ID, a sample used for each pairwise comparison, and days (Detailed sample information shown in Supplementary Table 2).

To more fully investigate the dynamics of the changes in the PKS from shared *B. vulgatus* and *B. uniformis* strains, it would be necessary to analyze longitudinal samples from healthy individuals taken at much shorter time periods. A previous study reported on the sampling of the fecal samples taken from healthy individuals at high-density (some are daily collected samples) followed by metagenomic sequencing for each individual^16^. Consistent with our previous analysis on the HMP data set, we found a shared *B. vulgatus* and *B. uniformis* strains as determined from a WSS analysis for each pair from 6 individuals over the times examined. We next conducted the PKS analysis on the 6 individuals to investigate differences in the shared *B. vulgatus* and *B. uniformis* strains (Figs. 2 and 3). For *B. vulgatus*, there were no shared PKS patterns observed from three individuals (AAD, AAN, and AAG) between the times that were examined, (*i.e.* AAD: 0 differences between 6 total pairs; AAN: 0 differences between 9 total pairs; AAG: 0 differences between 12 total pairs) (Fig. 2A–C). These results are consistent with the HMP and Raymond et al. data sets. In contrast, the remaining three individuals (AAP, AAI, and AAB) showed a clustering pattern with shared PKS at certain times (Fig. 3A–C). For example, AAP showed clusters of shared sample patterns between days 1 and 3 and between days 21 and 62 (4 of 12 pairs) (Fig. 3A). Individual AAI displayed a complex pattern in which a sample at day 2 became extinct from days 5–7 but reappeared on days 14, 21, and 28 before becoming extinct at later time points (4 of 15 pairs) (Fig. 3B). Analysis of individual AAB showed the most complex cluster patterns. For AAB, we found one pattern (the day 0 time point), with multiple times of extinction and reappearance (4 separate times) of different clusters (Fig. 3C). We also found two additional shared time points between days 8 and 33 and between days 31 and 32 (Fig. 3C). In total, we found 15 of 22 pairs were shared or repeated in this individual. Finally, we found that for all three individuals (AAP, AAI, and AAB) that the clusters of shared PKS patterns resolved to a unique pattern after time, indicating temporal cycling between the unique and cluster patterns in these individuals. A similar pattern was observed for *B. uniformis* showing that there were no shared PKS patterns observed from three individuals (AAG, AAI, and AAN), however the remaining two individuals (AAB and AAD) showed a clustering pattern with shared PKS at certain times (Supplementary Figs. 4 and 5).

### PKS analysis of patients hospitalized with COVID-19

**Figure 4:**
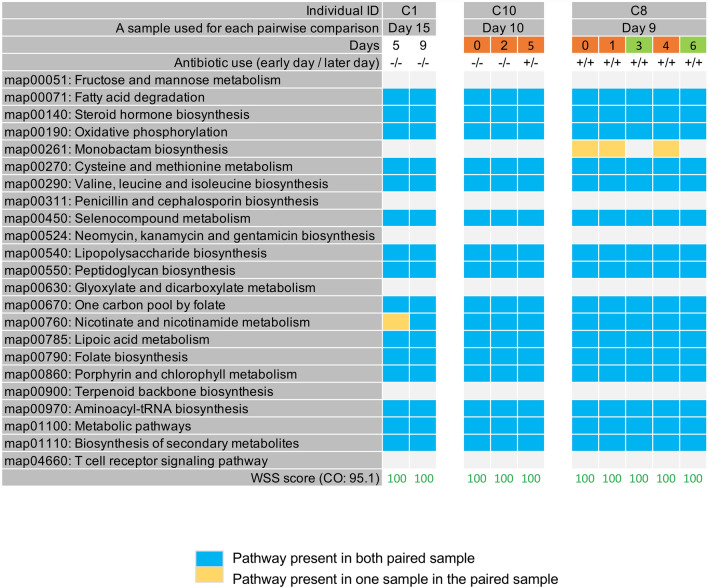
PKS results from hospitalized individuals with COVID-19. A total of 23 KEGG pathways were used to examine a pattern of presence/absence of KEGG metabolic pathways for *B. vulgatus* and the presence or absence of each KEGG pathway was observed by comparing each patient’s last day sample to every possible pair of the same patient’s samples. All patient samples were previously collected by Zuo et al. ^17^. The shared PKS result per patient was grouped into different color boxes. Each column in the table indicates individual ID, a sample used for each pairwise comparison, and days. (Detailed information provided in Supplementary Table 2). 5 additional patients’ PKS results are shown in Supplementary Fig. 2.

A recent paper used high-density fecal sampling and metagenomic sequencing to characterize the microbial community of hospitalized COVID-19 patients^17^. Using a data set of 8 patients with COVID-19 that had sufficient sequencing read depth we used the WSS analysis to determine no strain change occurred for the *B. vulgatus* during the stay in the hospital. For these same individuals, we then used the PKS analysis and found unique PKS patterns in 6 of the 8 patients (Fig. 4 and Supplementary Fig. 1). However, two individuals (C10 and C8) showed shared PKS clusters during the examined time points (Fig. 4). Individual C10 showed a pattern that was shown at a day 0 that was continued up to day 5 (Fig. 4). In contrast, in the C8, we found one pattern (the day 0 time point) that became extinct from day 3 but reappeared on day 4. In this individual, we also found another cluster on days 3 and 6 (Fig. 4). However, for these two patients, we did not see a resolution of the PKS clusters to a unique pattern in the times examined. For *B. uniformis*, one individual (C11) showed shared PKS clusters during the examined time points, however the remaining five individuals showed a unique pattern in the times examined (Supplementary Fig. 1).

## Discussion

In this study, we have utilized several previously published datasets to develop a new method that detects changes in the pattern of selected KEGG metabolic pathways for a selected strain. Our analysis provides new insights into the dynamics of microbial strain variation in healthy individuals and suggests that gut microbial communities under stress, such as those found in COVID-19 hospitalized patients, might be in a state of dysbiosis indicating the potential for the dominant strain to be outcompeted by a minor strain.

Disruptions of the gut microbial community could result in a dysbiosis leading to a strain variation that would, in turn, alter the community structure and impact the functions in metabolism and colonization resistance^15,18–20,25–27^. To detect microbial strain variation, our approach has been to use metagenomic DNA sequencing analysis coupled with informatics that can resolve the microbial community at a strain level^5,11^. This analysis works well with paired samples to investigate the question of whether strains are shared in an individual over time. However, we wanted to extend this analysis to look in greater detail at the shared strains to determine if we could delineate the dynamics of strain variation to identify indicators of an impending strain variation. To do this, in the current study, we have further characterized shared strain pairs by combining them with a new method to determine the pattern of presence/absence of KEGG metabolic pathways for shared strain (*i.e.* above the WSS cut-off value) between two paired samples. We focused on *B. vulgatus* and *B. uniformis* strains since they are predominant commensal in humans, possibly serving as a keystone species whose metabolic functions are important for the host^9,21–23^. We first developed this analysis by using an established data set that contained paired samples from a healthy individual. The HMP data set in which pairs from individuals taken at the time within 1 year that our previous WSS analysis had shown contained shared strains^5^. For this study, we first conducted the PKS analysis on the original sequence reads. To determine if we were analyzing the dominant *B. vulgatus* and *B. uniformis* strains in the community, we also performed subsampling on total sequence reads at various numbers. We then selected an optimized read number where the WSS analysis still detected related samples and the PKS gave a similar percentage of pairs with zero change compared with the original reads (Supplementary Fig. 1 and Supplementary Table 1). Thus, at this sequence read number (5 million) we were analyzing the dominant *B. vulgatus* and *B. uniformis* strains in the microbial community. We next applied our PKS analysis to examine a sample set from individuals where samples were collected at 0 and 90 days, with a dose of a single antibiotic in between those days that in some individuals resulted in transient strain change. We determined that 11 sample pairs still shared *B. vulgatus* strain from WSS analysis, however the PKS analysis showed that there were no KEGG pathways patterns shared between the sample pairs. It is important to note that for the PKS, we do not determine the use of the pathway function by the microbes. Essentially, these differences were distinguished between related strains as determined from the WSS analysis. Thus, the results of our studies that combine the WSS strain tracking with PKS provide new insights into the heterogeneity of human the individual specific microbe strain variants present within the human gut microbial strain community.

An explanation for the different (unique) PKS between paired samples observed at longer time points (*i.e.* within 1 year) might be in the resiliency of the healthy human microbial strain community to recover from disruptions^27^. To determine if strain variations in healthy individuals occurred, we selected a data set from six healthy individuals that had been sampled multiple times over a shorter time period^16^. While we did not find evidence for strain change using the WSS analysis during the time examined, we did find differences in the presence of unique and shared PKS between *B. vulgatus* and *B. uniformis*. We note that only one of these individuals, AAB, had shared PKS for both *B. vulgatus* and *B. uniformis*, indicating the possibility of a generalized dysbiosis in the microbial gut community environment. Given the greater numbers of the shared PKS, it is possible that *B. vulgatus* might be a more sensitive indicator of gut dysbiosis.

To extend these results, we next characterized a recent data set from COVID-19 hospitalized patients with mild to severe disease that had high-density fecal sampling over a short time period^17^. In these patients, four of the eight totals had received no antibiotics, with the remaining four given antibiotics during the hospital stay. Analysis of eight patients with the WSS analysis revealed that none of the patients had strain change in *B. vulgatus* over the time frame examined. For six patients, we only found unique PKS, although for five of the sample sets we were only able to analyze paired sample sets due to sequencing limitations. However, for two of the eight, we found clusters of shared PKS that during the time examined, did not transition to unique PKS as we found with the healthy individuals, although we recognize this could be due to limited sampling. Similarly, for *B. uniformis*, three individuals had unique patterns of PKS while two had clusters of PKS in the longitudinal data set; one of the eight patients had clusters of PKS. Collectively, these results highlight the benefit of high-density longitudinal sampling for the detailed analysis of the dynamics of shared gut microbial community strains. In some individuals, we found the oscillating between clustering of PKS and unique PKS giving new insights into the dynamics of temporal change within the normal microbial strain community^4^.

What might be the significance then of our identification of shared clusters of PKS over these short times in certain individuals with respect to actual strain change? One of the features of a complex biological system is that as it approaches a critical transition there is a slowing down of the intrinsic rates of change^28–32^. The system enters a condition that is related to autocorrelation (or serial correlation) where the patterns would be repeated between time points. It is possible that the shared PKS clusters represent a state of autocorrelation in the gut microbial strain community. These clusters could represent an early warning signal for a dysbiosis in which the dominant strain is replaced by a minor strain. The fluctuation between unique and shared clusters could be the result of environmental changes in the gastrointestinal tract environment and/or diet. Indeed, we note that the sample from the six healthy individuals who had the most observed autocorrelation with repeated PKS was from the oldest individual (AAB; 56 years old). The aging microbiome in combination with changes in environment or diet has been reported to be more susceptible to breakdowns in colonization resistance as reflected from colonization with pathogens^33^. Similarly, for two of the eight hospitalized COVID-19 patients, we found clusters of shared PKS suggesting these individuals’ microbial communities were under stress that could impact the normal functions in metabolism and, more importantly, colonization resistance that could be of great concern in a hospital environment^25,26^. We recognize though a limitation of our study is that, at this time, we cannot determine whether the dysbiosis microbial communities of the hospitalized COVID patients would have the resiliency to return to a normal pattern of unique PKS after time. Based on this concern though, we suggest that individualized monitoring of the gut microbial community in COVID-19 patients with multiple short term high-density sampling and analysis is appropriate to provide a personalized profile needed for evaluation and use of therapeutic interventions to maintain a healthy gut microbial community^34^.

## Materials and methods

### Publicly available data sets used in this study

In this study, we used 3 publicly available data sets for healthy individuals 1) pre-treated with iso-osmotic bowel wash^16^; 2) pre and post treated with a single antibiotic (cefprozil)^24^; and 3) from the NIH Human Microbiome Project (HMP)^8^. For Fukuyama et al., fecal samples from 6 individuals were collected pre and post mechanical bowel wash, and we selected only pre bowel wash samples for individuals. For the Raymond et al., fecal samples were collected from 18 individuals at three different time points: pre-treatment (Day 0), end of antibiotic treatment (Day 7), and 3 months post-treatment (Day 90), and we selected only Day 0 and Day 90 samples from 11 individuals for the analysis. For the HMP data set, 41 individuals which were previously used to establish our WSS analysis were selected for the analysis^5^. In addition, we used 1 publicly available data set from patients with COVID-19^17^. For Zuo et al., fecal samples from 15 patients with COVID-19 were collected during the time of hospitalization and we selected 8 patients’ samples to run the analysis. All data sets used in this study were summarized in Supplementary Table 2.

A total of 6,390,986,972 metagenomic sequencing reads were downloaded from the four public data sets; 359,276,476 reads from the Fukuyama et al., 1,500,234,831 from the Raymond et al., 4,124,040,250 from the HMP data set, and 407,435,415 from the Zuo et al. (Supplementary Table 2). Quality control steps include removing any human reference genome (hg19) using bowtie2 (version 2.3.4.3) with default parameters^35^, and filtering short sequences (sequence length < 50 bases) and low quality reads (sliding window of 50 bases having a QScore < 20) using Trimmomatic (version 0.36)^36^.

### Strain-tracking analysis using WSS

For the HMP data set, we have previously applied our WSS analysis to investigate the strain relatedness for each individual between longitudinal fecal samples and establish each species’ WSS cut-off value for relatedness^5^. In this study, we used 1) randomly subsampled the HMP data set at various reads (2.5, 5, and 10 million reads); and 2) the original sequence reads (average value of ~ 50 million reads) to run the WSS analysis for strain relatedness as well as to validate our PKS method. All of the individual samples from the remaining data sets (Fukuyama et al., Raymond et al., and Zuo et al., data sets) were randomly subsampled (seed = 1000) to 5 million reads with seqtk (version 1.3) (https://github.com/lh3/seqtk). The quality control steps were then applied to the subsampled sequence reads. For Fukuyama et al. data set^16^, we have investigated strain relatedness for each individual by comparing all available longitudinal samples to the last available sample, those were collected before the bowel wash procedure was performed. The strain-tracking analysis was also applied for the Raymond et al. data set^24^ to examine strain relatedness for each individual between pre (Day 0) and post antibiotic treatment (Day 90). From the Zuo et al. data set^17^, the strain-tracking analysis was performed for each COVID-19 patient by comparing all available longitudinal samples to the last available sample.

Our WSS strain-tracking analysis was then conducted on these data sets and the full details of the analysis procedure can be found in our previously published papers^5,11,13,14,37,38^. All codes implemented in the WSS were deposited and are available at https://github.com/hkoo87/mgSNP_2.

### Description of the methodology used for PKS analysis

In this study, as an example, we have selected *B. vulgatus* and *B. uniformis* to investigate patterns of presence/absence of selected KEGG metabolic pathways (PKS). To do this, we first included the selected species alignments from each sample’s ‘realigned.bam’ file, which is one of the output files generated from the WSS analysis including each sample SNVs for each given reference sequence using samtools view -b option^39,40^. The filtered alignments were then sorted, indexed using samtools sort and index function, respectively with default parameter^39,40^ and the resultant bam file was converted to FASTQ format using BEDTools with bamtofastq function with default parameter^41^. All these steps can be done using the ‘PKS_bamtofastq.sh’ script included in the PKS method. Second, the converted FASTQ file was assembled using MEGAHIT with default parameter (version 1.1.3) and the resultant ‘final.contigs.fa’ file for each sample was selected for annotation^42^. This step can be accessed using the ‘PKS_megahit_run.sh’ script included in the PKS method. Lastly, each sample’s ‘final.contigs.fa’ was annotated using Prokka (version 1.14.0) with –cpus 0 –addgenes –metagenome –mincontiglen 1 parameters^43^. Then, ‘eC_number’ was grepped from one of Prokka’s output files, ‘.gff’, using grep function. Each ‘eC_number’ was then annotated against KEGG database using MinPath (version 1.4) and the resultant ‘map ID’ was selected based on the ‘minpath’ value of 1^44^. All annotation steps can be done using the ‘PKS_Prokka_Minpath.sh’ script included in the PKS method. All codes used for the PKS analysis were deposited and are available at https://github.com/hkoo87/PKS.

### Validation of the methodology for PKS analysis

In this study, we have used the HMP data set to validate our PKS method and establish a list of KEGG pathways, specifically for *B. vulgatus* and *B. uniformis* by random subsampling of sequence reads at 2.5, 5, and 10 million. All subsampling step was conducted using seqtk (version 1.3) (https://github.com/lh3/seqtk). For each subsampling step, WSS analysis along with the PKS method was conducted for each pairwise comparison to monitor changes in the WSS score as well as PKS result. Both WSS and PKS method was first used for each pairwise comparison using the original number of sequences reads. To establish a list of KEGG pathways, all pathways observed from the PKS analysis of the original sequence reads were combined; pairs that showed an unrelated WSS score were excluded. All observed KEGG pathways were also combined from the PKS analysis of each subsampled sequence reads also excluding pairs that had unrelated WSS scores. For *B. vulgatus*, a KEGG list including a total of 23 pathways was established from the 5 million subsampled sequence reads and used as a standard list to compare PKS analysis results when other data sets analyzed with PKS due to those were subsampled to 5 million reads in this study. For *B. uniformis*, a total of 40 pathways were included in the KEGG list.

## Supplementary Information

Supplementary Information 1.

Supplementary Information 2.

Supplementary Information 3.

## Data Availability

The original sequencing data sets used in this study were downloaded from the NCBI under accession numbers, PRJNA388263 for Fukuyama et al., and PRJNA624223 for Zuo et al.; from the European Nucleotide Archive under accession number, PRJEB8094 for Raymond et al.; from https://portal.hmpdacc.org/ for the HMP data set.

## References

[1] Sekirov I, Russell SL, Antunes LCM, Finlay BB (2010). Gut microbiota in health and disease. Physiol. Rev..

[2] Vujkovic-Cvijin I (2020). Host variables confound gut microbiota studies of human disease. Nature.

[3] David LA (2014). Host lifestyle affects human microbiota on daily timescales. Genome Biol..

[4] Priya S, Blekhman R (2019). Population dynamics of the human gut microbiome: change is the only constant. Genome Biol..

[5] Kumar R (2017). Identification of donor microbe species that colonize and persist long term in the recipient after fecal transplant for recurrent Clostridium difficile. NPJ Biofilms Microbiomes.

[6] Segata N (2018). On the road to strain-resolved comparative metagenomics. MSystems.

[7] Truong DT, Tett A, Pasolli E, Huttenhower C, Segata N (2017). Microbial strain-level population structure and genetic diversity from metagenomes. Genome. Res..

[8] Methé BA (2012). A framework for human microbiome research. Nature.

[9] Schloissnig S (2013). Genomic variation landscape of the human gut microbiome. Nature.

[10] Franzosa EA (2015). Identifying personal microbiomes using metagenomic codes. Proc. Natl. Acad. Sci..

[11] Koo H, Hakim JA, Crossman DK, Lefkowitz EJ, Morrow CD (2019). Sharing of gut microbial strains between selected individual sets of twins cohabitating for decades. PLoS ONE.

[12] Kumar R (2018). New microbe genomic variants in patients fecal community following surgical disruption of the upper human gastrointestinal tract. Human Microbiome J..

[13] Koo H, Morrow CD (2020). Perturbation of the human gastrointestinal tract microbial ecosystem by oral drugs to treat chronic disease results in a spectrum of individual specific patterns of extinction and persistence of dominant microbial strains. PLoS ONE.

[14] Koo H (2019). Individualized recovery of gut microbial strains post antibiotics. NPJ Biofilms Microbiomes.

[15] Garud NR, Good BH, Hallatschek O, Pollard KS (2019). Evolutionary dynamics of bacteria in the gut microbiome within and across hosts. PLoS Biol..

[16] Fukuyama J (2017). Multidomain analyses of a longitudinal human microbiome intestinal cleanout perturbation experiment. PLoS Comput. Biol..

[17] Zuo T (2020). Alterations in gut microbiota of patients with COVID-19 during time of hospitalization. Gastroenterology.

[18] Sharon I (2013). Time series community genomics analysis reveals rapid shifts in bacterial species, strains, and phage during infant gut colonization. Genome Res..

[19] Greenblum S, Carr R, Borenstein E (2015). Extensive strain-level copy-number variation across human gut microbiome species. Cell.

[20] Zhu A, Sunagawa S, Mende DR, Bork P (2015). Inter-individual differences in the gene content of human gut bacterial species. Genome Biol..

[21] Wexler HM (2007). Bacteroides: the good, the bad, and the nitty-gritty. Clin. Microbiol. Rev..

[22] Kamada N, Chen GY, Inohara N, Núñez G (2013). Control of pathogens and pathobionts by the gut microbiota. Nat. Immunol..

[23] Wexler AG, Goodman AL (2017). An insider's perspective: Bacteroides as a window into the microbiome. Nat. Microbiol..

[24] Raymond F (2016). The initial state of the human gut microbiome determines its reshaping by antibiotics. ISME J..

[25] Buffie CG, Pamer EG (2013). Microbiota-mediated colonization resistance against intestinal pathogens. Nat. Rev. Immunol..

[26] Sorbara MT, Pamer EG (2019). Interbacterial mechanisms of colonization resistance and the strategies pathogens use to overcome them. Mucosal Immunol..

[27] Faust K, Lahti L, Gonze D, De Vos WM, Raes J (2015). Metagenomics meets time series analysis: unraveling microbial community dynamics. Curr. Opin. Microbiol..

[28] Dai L, Vorselen D, Korolev KS, Gore J (2012). Generic indicators for loss of resilience before a tipping point leading to population collapse. Science.

[29] Dakos V (2012). Methods for detecting early warnings of critical transitions in time series illustrated using simulated ecological data. PLoS ONE.

[30] Scheffer M (2009). Early-warning signals for critical transitions. Nature.

[31] Dakos V (2008). Slowing down as an early warning signal for abrupt climate change. Proc. Natl. Acad. Sci..

[32] Scheffer M (2012). Anticipating critical transitions. Science.

[33] Kim S, Jazwinski SM (2018). the gut microbiota and healthy aging: A mini-review. Gerontology.

[34] Tursi A, Alfredo P (2020). Intestinal microbiome modulation during COVID-19: Another chance to manage the disease?. Gastroenterology.

[35] Langmead B, Salzberg SL (2012). Fast gapped-read alignment with Bowtie 2. Nat. Methods.

[36] Bolger AM, Lohse M, Usadel B (2014). Trimmomatic: A flexible trimmer for Illumina sequence data. Bioinformatics.

[37] Koo, H., Crossman, D. K. & Morrow, C. D. Strain tracking to identify individualized patterns of microbial strain stability in the developing infant gut ecosystem. *Front. Pediatr.* **8** (2020). 10.3389/fped.2020.549844 PMC7555834 33102406

[38] Koo H (2020). An individualized mosaic of maternal microbial strains is transmitted to the infant gut microbial community. Royal Soc. Open Sci..

[39] Li H (2009). The sequence alignment/map format and SAMtools. Bioinformatics.

[40] Li H (2011). A statistical framework for SNP calling, mutation discovery, association mapping and population genetical parameter estimation from sequencing data. Bioinformatics.

[41] Quinlan AR, Hall IM (2010). BEDTools: a flexible suite of utilities for comparing genomic features. Bioinformatics.

[42] Li D, Liu C-M, Luo R, Sadakane K, Lam T-W (2015). MEGAHIT: an ultra-fast single-node solution for large and complex metagenomics assembly via succinct de Bruijn graph. Bioinformatics.

[43] Seemann T (2014). Prokka: rapid prokaryotic genome annotation. Bioinformatics.

[44] Ye Y, Doak TG (2009). A parsimony approach to biological pathway reconstruction/inference for genomes and metagenomes. PLoS Comput. Biol..

