# Supplementary material for: Early indicators of microbial strain dysbiosis in the human gastrointestinal microbial community of certain healthy humans and hospitalized COVID-19 patients
Source: Sci Rep. 2022 Apr 21;12:6562. doi: 10.1038/s41598-022-10472-w (PMC9022020; doi:10.1038/s41598-022-10472-w)
Supplement: Supplementary file 2 — Supplementary Information 2. [file 41598_2022_10472_MOESM2_ESM.pdf]

# Early indicators of microbial strain dysbiosis in the human gastrointestinal microbial community of certain healthy humans and hospitalized COVID-19 patients

Hyunmin Koo[1*], and Casey D. Morrow[2*]

[1]Department of Genetics
Hugh Kaul Precision Medicine Institute
University of Alabama at Birmingham
Birmingham, Alabama, United States of America

[2]Department of Cell, Developmental and Integrative Biology
Hugh Kaul Precision Medicine Institute
University of Alabama at Birmingham
Birmingham, Alabama, United States of America

## Description of Supplementary Information:

Supplementary Information file includes Supplementary Figures 1-6.

## Supplementary Figures

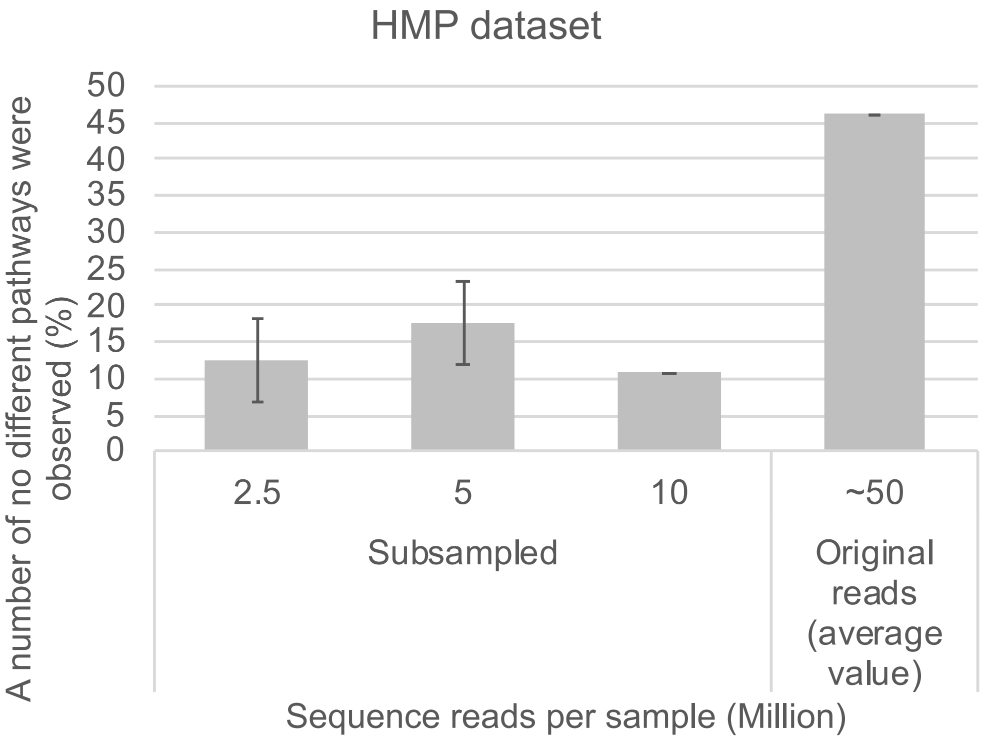


**Supplementary Figure 1: PKS analysis on the HMP data set with and without subsampling sequence reads.**
To further characterize the PKS analysis, we have conducted the PKS analysis on the HMP data set with no subsampling process involved and with a random subsampling process involved at 2.5, 5, and 10 million sequence reads. For the 2.5 and 5 million reads, we repeated the random subsampling process two more times to detect any variations in the WSS score or PKS. Detailed information is shown in **Supplementary Table 1**. We also did a same analysis (random subsampling processes and no subsampling process) for *B. uniformis*. Overall, for 5 million reads, an average value of 3 of the 24 sample pairs (12.5%) had 0 changes in the compared KEGG pathways and the remaining 21 pairs (87.5%) had no shared patterns for *B. uniformis*. For 10 million reads, 24.14% sample pairs (7 of the 29 sample pairs) had 0 changes in the compared KEGG pathways and for original reads, 37.14% (13 of the 35 sample pairs) sample pairs had 0 changes in the compared KEGG pathways.

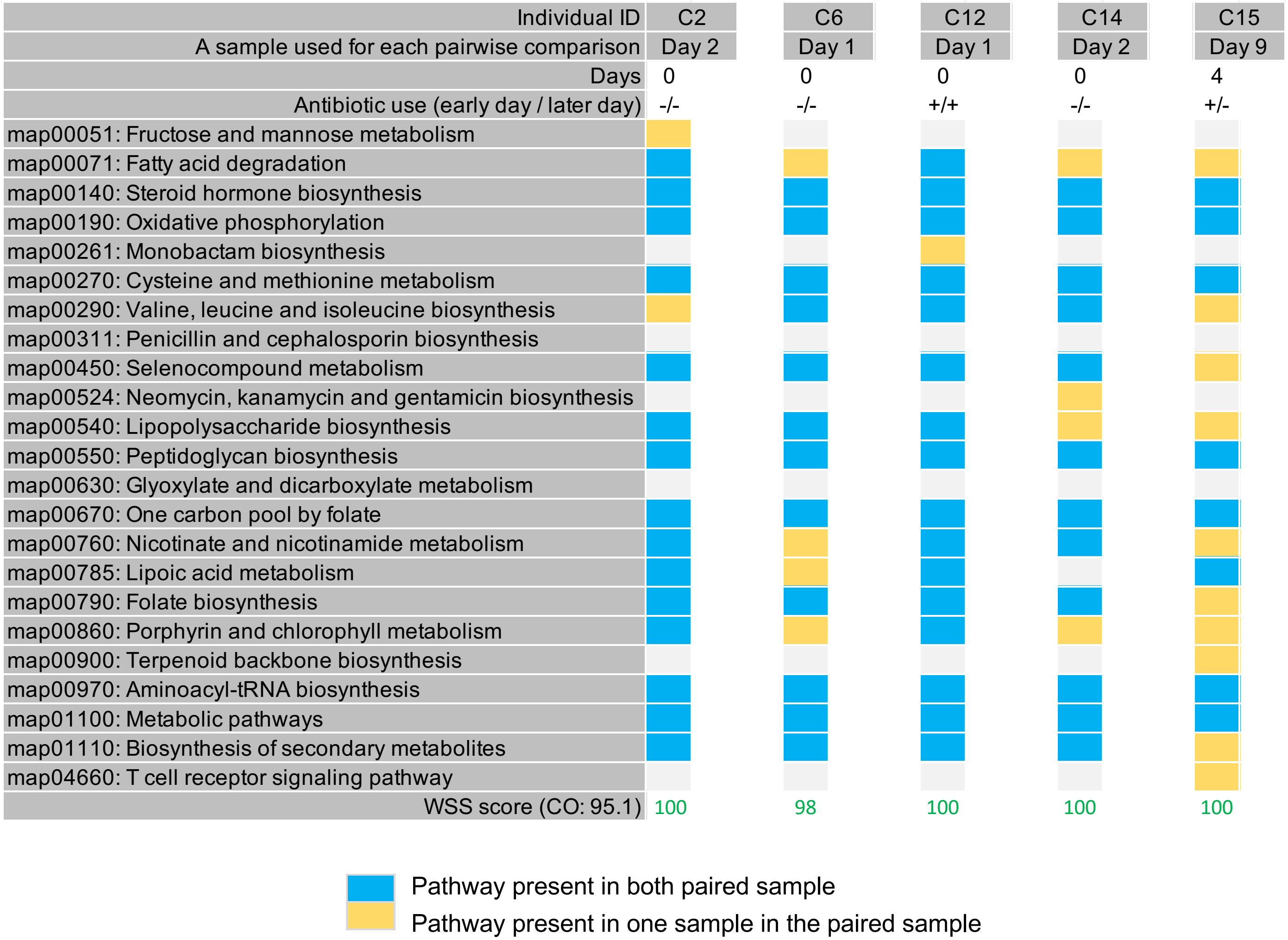


**Supplementary Figure 2: Summarized PKS results for Zuo et al. from 5 patients.**
A total of 23 KEGG pathways were used to investigate a pattern of presence/absence of KEGG metabolic pathways for *B. vulgatus* and the presence or absence of each KEGG pathway was observed by comparing each patient's last day sample to every possible pair of the same patient's samples. All patient samples were previously collected by Zuo et al. [17]. The summarized PKS result per patient was grouped into different color boxes (colors elaborated in a label). Each column in the table indicates individual ID, a sample used for each pairwise comparison, days, and antibiotic use (Detailed information provided in **Supplementary Table 2**).

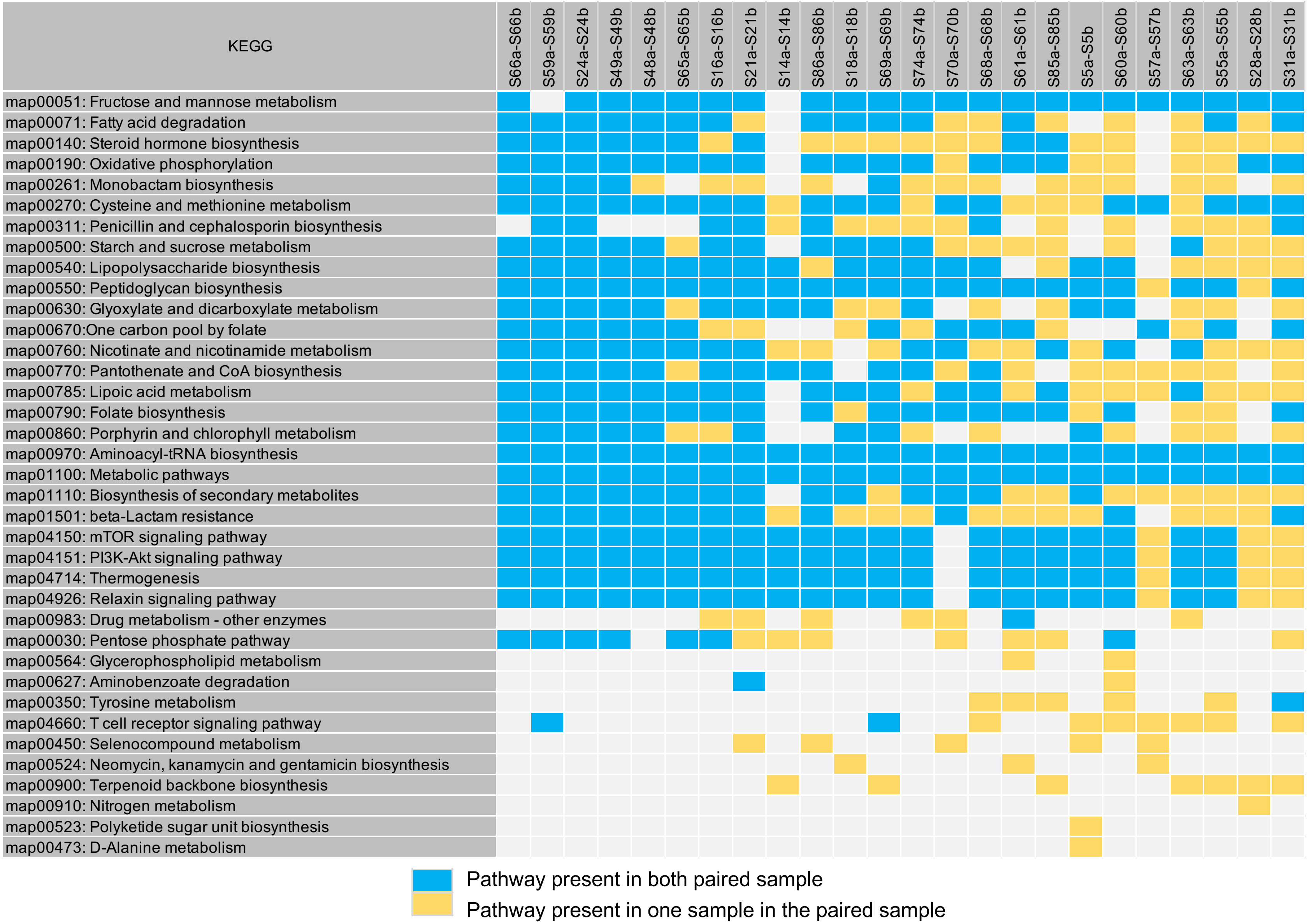


**Supplementary Figure 3:** ***B. uniformis*** **PKS results for HMP data set.**
A total of 37 KEGG pathways were used to investigate a pattern of presence/absence of metabolic pathways for *B. uniformis*. The summarized PKS result were grouped into different color boxes (colors scheme presented in the figure).

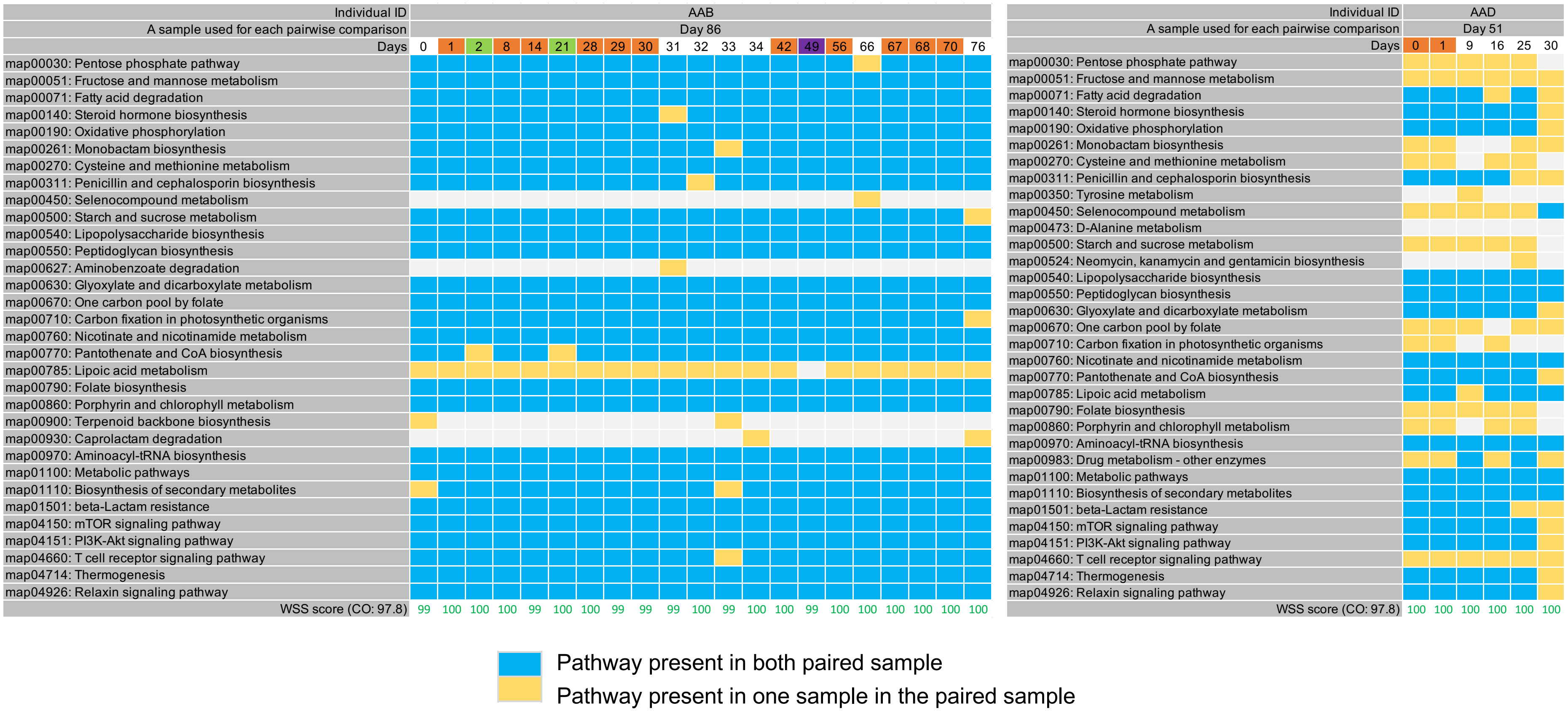


**Supplementary Figure 4: *B. uniformis* shared PKS pattern for high density sample.**
The KEGG pathways were used to find the presence/absence of KEGG metabolic pathways for *B. uniformis* by paring each individual's last day sample to every possible pair of the same individual's samples. All samples were previously collected by Fukuyama et al.[16]. The summarized PKS result per individual was grouped into different color boxes.

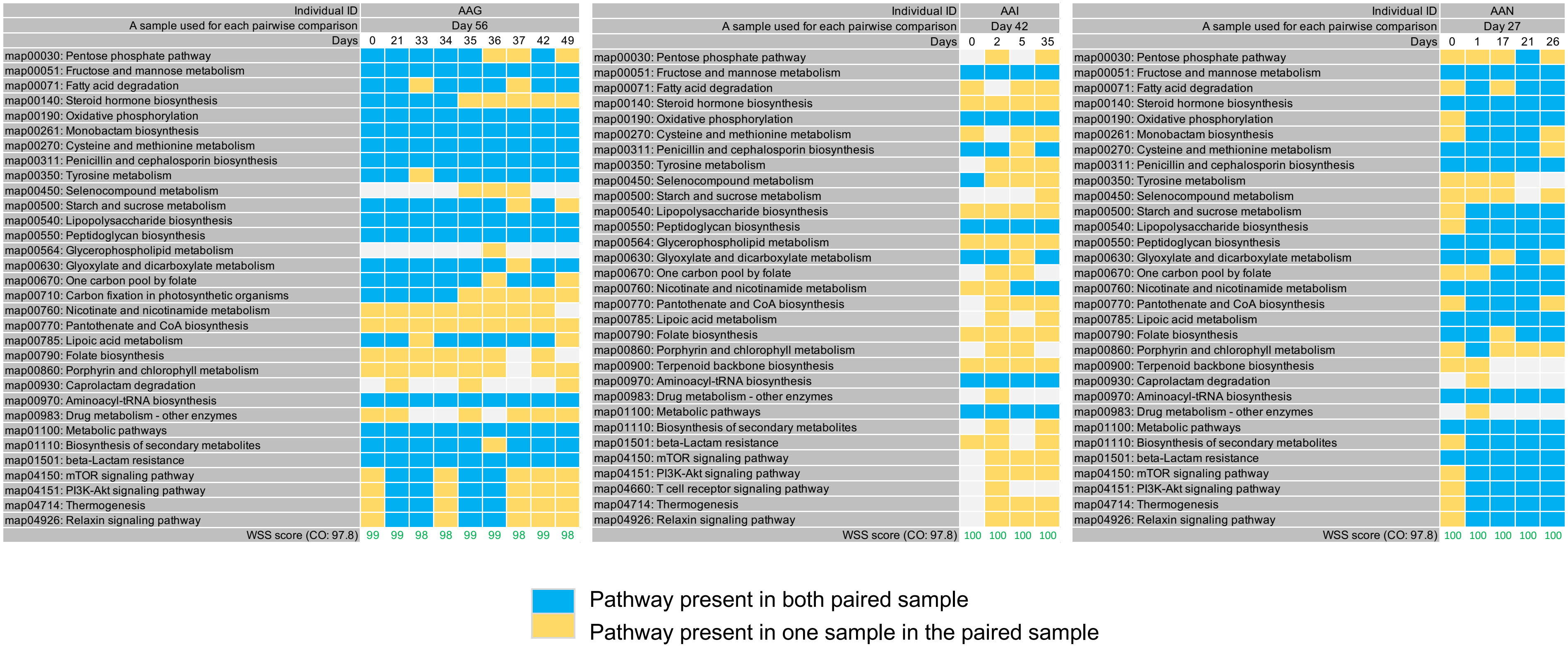


**Supplementary Figure 5: *B. uniformis* unique PKS pattern for high density sample.**
The KEGG pathways were used to find the presence/absence of KEGG metabolic pathways for *B. uniformis* by paring each individual's last day sample to every possible pair of the same individual's samples. All samples were previously collected by Fukuyama et al.[16]. The summarized PKS result per individual was grouped into different color boxes.

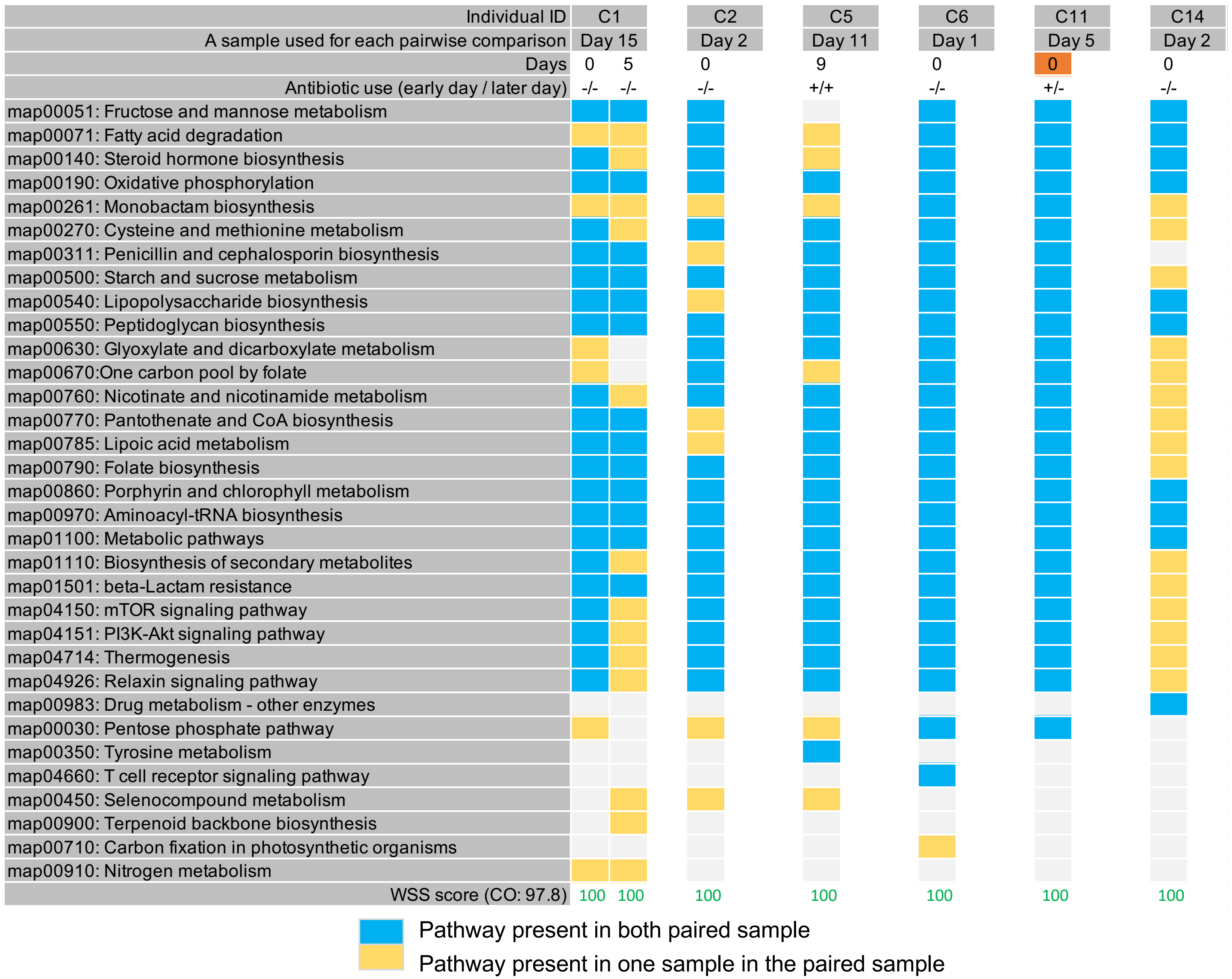


**Supplementary Figure 6: *B. uniformis* PKS results from hospitalized individuals with COVID-19.**
The KEGG pathways were used to examine a pattern of presence/absence of KEGG metabolic pathways for *B. uniformis.* All patient samples were previously collected by Zuo et al. [17]. The shared PKS result per patient was grouped into different color boxes.
